# Picosecond 755-nm alexandrite laser for labial lentigines in Peutz-Jeghers syndrome: A procedural case report

**DOI:** 10.1016/j.jdcr.2025.11.051

**Published:** 2025-12-16

**Authors:** Pedro Simões Farinha, Maria Paiva-Lopes, Alexandre João

**Affiliations:** a Dermatology Department, Hospital de S. José, Unidade Local de Saúde São José, Lisbon, Portugal; b Dermatology Division, Centro Clínico Académico de Lisboa, Lisbon, Portugal; c Department of Dermatology, NOVA Medical School (NMS FCM), Universidade NOVA de Lisboa, Lisbon, Portugal; d Derma 360, Lisbon, Portugal

**Keywords:** buccal lentigines, cosmetic dermatology, labial lentigines, Peutz-Jeghers syndrome, picosecond alexandrite laser, procedural dermatology

## Introduction

Peutz–Jeghers syndrome (PJS) is an autosomal dominant disorder caused by germline mutations in *STK11/LKB1*, a tumor suppressor serine–threonine kinase that regulates AMPK family members, cell polarity, metabolism, and apoptosis. Biallelic inactivation underlies the phenotype of GI hamartomatous polyps and mucocutaneous pigmentation. Mucocutaneous macules occur in >95% of patients, most commonly on lips/perioral skin and buccal mucosa; lesions on skin often fade after puberty, whereas buccal mucosal macules persist. Histologically, pigmentation reflects increased basal melanin with superficial dermal melanophages; melanocyte density is normal to slightly increased, akin to lentigo simplex.^1^^,^^2^

Although the histologic basis of pigmentation is well characterized, the molecular mechanism remains poorly elucidated. *STK11/LKB1* loss alters cellular growth regulatory pathways (AMPK/mTOR, Wnt, TGF-β) and may secondarily influence melanogenesis, but experimental data specifically addressing mucocutaneous pigmentation in PJS are lacking.^1^^,^^2^

The psychosocial burden is nontrivial: a quantitative study documented mild depression, worse mental health perception, and altered life decisions among patients with PJS.^3^

Cosmetic treatment is justified in selected adolescents/adults. Q-switched (QS) lasers—alexandrite 755 nm and Nd:YAG 532 nm—have shown consistent efficacy. In 43 patients, QS alexandrite achieved ≥75% clearance after ∼3 sessions^4^; QS Nd:YAG yielded excellent responses in most of 11 patients after ∼3-4 sessions.^5^ A 2022 review (81 patients) found lasers/IPL broadly effective, with QS alexandrite most used.^6^

Evidence for picosecond (ps) 755-nm alexandrite remains limited to 2 reports: a Spanish case^7^ and a two-patient Chinese series,^8^ both showing rapid, high-grade clearance with minimal adverse effects. Ps pulses enhance photoacoustic pigment fragmentation with reduced thermal diffusion, potentially lowering PIH risk and session number compared with nanosecond QS devices.

## Case report

**Fig 1:**
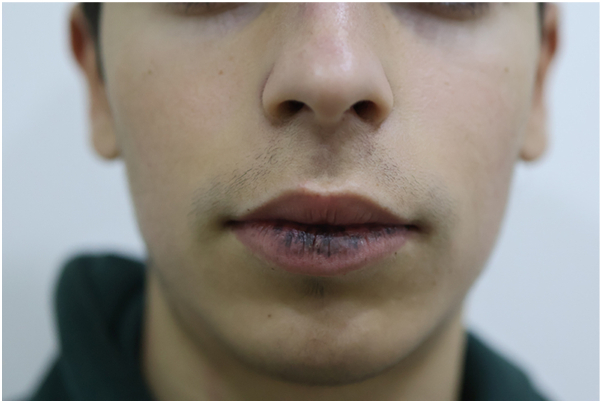
Multiple *brown macules* on the lower lip before treatment, consistent with labial lentigines in Peutz-Jeghers syndrome.

**Fig 2:**
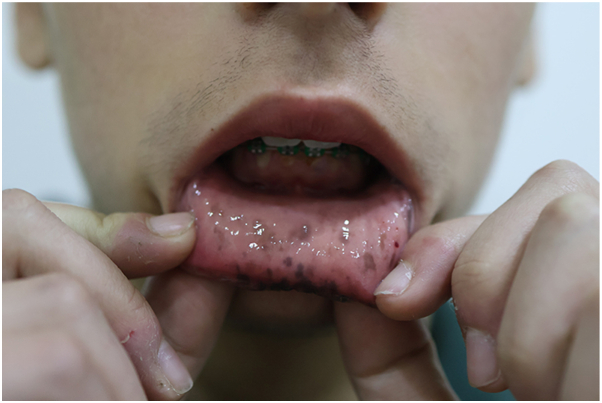
Multiple pigmented macules on the buccal mucosa before treatment.

A 15-year-old male with genetically confirmed PJS (positive family history in his father) presented with cosmetically distressing lentiginosis of the lower lip and buccal mucosa (Fig 1, Fig 2), causing social withdrawal and heightened self-consciousness.

After written consent, a test spot was performed with a picosecond 755-nm alexandrite platform (PicoZoom): **2.1 J/cm^2^, 2.5 Hz, 3.5-mm spot**, without adverse effects. The patient reported only minimal discomfort during treatment, described as a transient snapping sensation. No anesthesia was required, and the sessions were well tolerated without distress. Three subsequent treatments were delivered at **2.1-2.2 J/cm^2^, 2.5 Hz, 3.3-mm spot,** with 6-week intervals between sessions. Clinical endpoints included immediate macular darkening with faint whitening (“frosting”). Postprocedure care consisted of topical fusidic acid.

**Fig 3:**
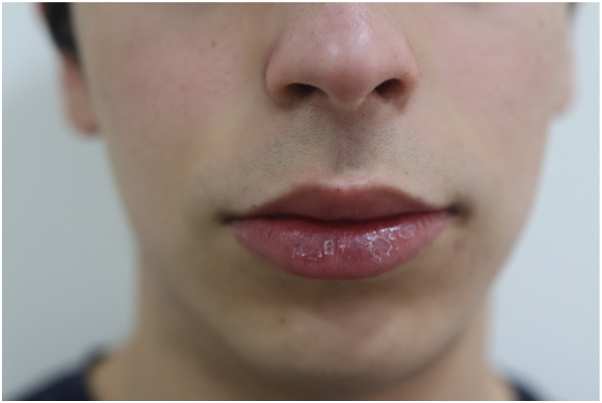
Marked clearance of lentigines on the lower lip after 3 sessions with the picosecond 755-nm alexandrite laser, documented at 6-month follow-up.

**Fig 4:**
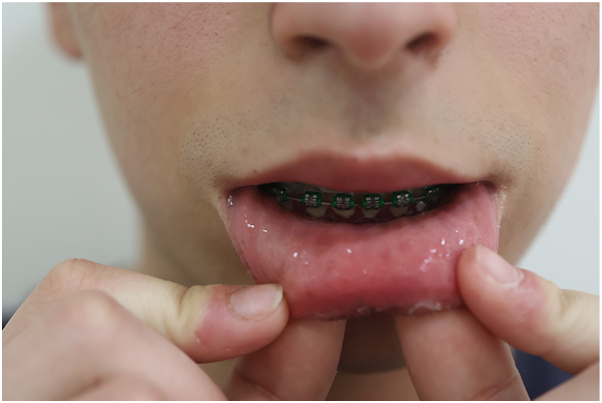
Near-complete clearance at 6-month follow-up, following the 3 and final treatment session.

Outcome: progressive, marked clearance was observed at 3 m (after the second session), with near-complete resolution at 6 m (after the third session) (Fig 3, Fig 4). Although no validated quality of life instrument was applied, both patient and caregivers reported a subjective improvement in emotional well-being and social confidence. No PIH, scarring, or recurrence were noted during follow-up.

## Discussion

PJS-associated lentiginosis mirrors lentigo-like basal layer hypermelanosis with melanophages in the superficial dermis; while medically benign, persistent oral lesions confer visible stigma. Documented psychosocial morbidity supports offering aesthetic intervention to suitable adolescents.^3^

Comparative evidence favors lasers targeting melanin. QS alexandrite (755 nm) exploits selective photothermolysis; in 43 patients, ≥75% clearance typically required ∼3 sessions.^4^ QS Nd:YAG (532 nm) achieved excellent clearance in ∼73% of 11 patients after a mean 3.6 sessions,^5^ with repeated treatments improving outcomes. A systematic review across 81 patients treated with lasers/IPL confirmed broad efficacy and acceptable safety, with QS alexandrite the most frequently reported platform.^6^

Picosecond alexandrite introduces ultrashort pulse durations (hundreds of ps), generating higher peak powers and a predominant photoacoustic mechanism that pulverizes melanosomes with less collateral thermal injury. This biophysical profile is associated with faster clearance and theoretically lower PIH/downtime relative to nanosecond QS exposure. To date, only 2 published ps-alexandrite reports in PJS exist: Agud-Dios et al (2022),^7^ oral lentiginosis treated successfully with 755-nm ps alexandrite; and Zeng et al (2021),^8^ 2 adolescents achieving ≥75–≈100% clearance after 1-2 sessions without recurrence at 12 m. Our case aligns with these data, demonstrating substantial clearance in 3 sessions and excellent tolerance.

Limitations include the single-patient design, short follow-up, and absence of standardized ps parameters. Nevertheless, convergence of results across QS series, a systematic review, and the 2 ps reports—plus our experience—supports ps 755-nm alexandrite as a rational, well-tolerated option for PJS oral/labial lentiginosis when psychosocial impact is significant.

## Conclusion

Picosecond 755-nm alexandrite laser achieved safe, rapid esthetic improvement of labial/buccal lentiginosis in a PJS adolescent, consistent with sparse published evidence. Controlled studies are warranted to refine parameters, durability, and comparative effectiveness versus QS platforms.

## Conflicts of interest

None disclosed.

## References

[1] McGarrity T.J., Amos C.I. (2006). Peutz-Jeghers syndrome: clinicopathology and molecular alterations. Cell Mol Life Sci.

[2] Zbuk K.M., Eng C. (2007). Hamartomatous polyposis syndromes. Nat Clin Pract Gastroenterol Hepatol.

[3] Woo A., Sadana A., Mauger D.T., Baker M.J., Berk T., McGarrity T.J. (2009). Psychosocial impact of Peutz-Jeghers syndrome. Fam Cancer.

[4] Li Y., Tong X., Yang J., Yang L., Tao J., Tu Y. (2012). Q-switched alexandrite laser for facial/labial lentigines in PJS. Photodermatol Photoimmunol Photomed.

[5] Ge Y., Jia G., Lin T. (2015). Q-switched Nd:YAG (532 nm) for labial lentigines in PJS. J Dtsch Dermatol Ges.

[6] Medeiros Y.L., Faria L.V., Chandretti P.C., Mainenti P. (2022). Lasers and IPL for labial lentigines in PJS: review. Dermatol Ther.

[7] Agud-Dios M., Perandones-Gonzalez H., Fernández-Domper L., Dominguez Santas M., Boixeda de Miquel P. (2022). 755-nm picosecond alexandrite for oral lentiginosis in PJS. Lasers Surg Med.

[8] Zeng R., Wu Q., Gu L., Lin T. (2021). 755-nm picosecond alexandrite for oral spots in PJS (2 adolescents). Indian J Dermatol Venereol Leprol.

